# Genes Encoding Multiple Modulators of the Immune Response Are Methylated in the Prostate Tumor Microenvironment of African Americans

**DOI:** 10.3390/cancers17142399

**Published:** 2025-07-19

**Authors:** Vinay Kumar, Tara Sinta Kartika Jennings, Lucas Ueta, James Nguyen, Liankun Song, Michael McClelland, Weiping Chu, Michael Lilly, Michael Ittmann, Patricia Castro, Arash Rezazadeh Kalebasty, Dan Mercola, Omid Yazdanpanah, Xiaolin Zi, Farah Rahmatpanah

**Affiliations:** 1 Department of Pathology and Laboratory Medicine, University of California, Irvine, CA 92697, USA; kuvinay97@ucla.edu (V.K.); tarasj@uci.edu (T.S.K.J.); uetal@uci.edu (L.U.); james.nguyen@students.jefferson.edu (J.N.); dmercola@hs.uci.edu (D.M.); 2 Department of Urology, University of California, Irvine, CA 92697, USA; liankuns@hs.uci.edu (L.S.); xzi@uci.edu (X.Z.); 3 Department of Microbiology and Molecular Genetics, University of California, Irvine, CA 92697, USA; mmcclell@uci.edu (M.M.); weipingc@uci.edu (W.C.); 4 Department of Medicine, Division of Hematology and Oncology, Medical University of South Carolina (MUSC), Charleston, SC 29425, USA; lillym@musc.edu; 5 Department of Pathology and Immunology, Baylor College of Medicine, Houston, TX 77030, USA; mittmann@bcm.edu (M.I.); pcastro@bcm.edu (P.C.); 6 Division of Hematology and Oncology, University of California, Irvine, CA 92697, USA; arez@hs.uci.edu (A.R.K.); oyazdanp@hs.uci.edu (O.Y.)

**Keywords:** prostate cancer, African American, European American, tumor microenvironment, DNA methylation

## Abstract

**Background/Objectives**: Prostate cancer (PCa) is diagnosed at an earlier median age, more advanced stage, and has worse clinical outcomes in African American (AA) men compared to European Americans (EA). **Methods**: To investigate the role of aberrant DNA methylation in tumor-adjacent stroma (TAS), methyl binding domain sequencing (MBD-seq) was performed on AA (*n* = 17) and EA (*n* = 15) PCa patients. This was independently confirmed using the long interspersed nuclear element-1 (LINE-1) assay. Pathway analysis was performed on statistically significantly differentially methylated genes for AA and EA TAS. DNA methylation profiles of primary cultured AA and EA carcinoma-associated fibroblasts (CAFs) were compared with AA and EA TAS. AA and EA CAFs were treated with demethylating agent 5-Azacytidine (5-AzaC). **Results**: AA TAS exhibited higher global DNA methylation than EA TAS (*p*-value < 0.001). Of the 3268 differentially methylated regions identified (DMRs, *p*-value < 0.05), 85% (2787 DMRs) showed increased DNA methylation in AA TAS, comprising 1648 genes, of which 1379 were protein-coding genes. Based on DNA methylation levels, two AA subgroups were identified. Notably, AA patients with higher DNA methylation were predominantly those with higher Gleason scores. Pathway analysis linked methylated genes in AA TAS to several key signaling pathways (*p*-value < 0.05), including immune response (e.g., IL-1, IL-15, IL-7, IL-8, IL-3, and chemokine), Wnt/β-catenin, androgen, PTEN, p53, TGF-β, and circadian clock regulation. A total of 168 concordantly methylated genes were identified, with 109 genes (65%) showing increased methylation in AA CAFs and TAS (*p*-value < 0.05). Treatment with 5-AzaC significantly reduced DNA methylation of concordant genes in AA CAFs (*p*-value < 0.001). **Conclusions**: These findings suggest a distinct stromal methylome in AA, providing a foundation for integrating demethylating agents into standard therapies. This approach targets the tumor microenvironment, potentially addressing PCa disparities in AA men.

## Simple Summary

African American (AA) men with prostate cancer (PCa) have a higher incidence and twice the mortality compared to European American (EA) men. Aberrant DNA methylation in tumor-adjacent stroma (TAS) plays a crucial role in prostate cancer development. We investigated the differences in stromal DNA methylation between AA and EA PCa patients. This study has potential to identify stromal markers that can improve diagnosis and prognosis, as well as provide new therapeutic targets.

## 1. Introduction

Prostate cancer (PCa) is a multi-factorial disease, and identification of the drivers of prostate cancer development across various ethnic groups remains an active area of research. Generally, American patients of African ancestry (AA) tend to have worse clinical outcomes than their European American counterparts (EA), even after accounting for disparities in access to and quality of care [1]. African Americans with PCa typically present with higher prostate-specific antigen (PSA) levels and tumor volumes at the time of diagnosis [2]. They also face early age onset, increased mortality, shorter survival times, and higher likelihood of disease progression [2].

A growing body of research supports the hypothesis that there are genomic differences between men of different ancestries that contribute to health disparities in PCa, including gene expression levels, unique polymorphisms, and DNA methylation patterns [3].

The tumor microenvironment, particularly stromal cells adjacent to the tumor, play a crucial role in mediating PCa tumorigenesis [4], and is emerging as a potential target for cancer therapy in solid tumors [5]. The stromal component of the tumor microenvironment consists of both acellular and cellular elements. The acellular component primarily includes the extra cellular matrix (ECM), which provides structural support to surrounding cells. The dense ECM can create a barrier that impedes immune cell infiltration, contributing to an immunosuppressive tumor microenvironment (reviewed in [6,7]). The cellular components comprise cancer-associated fibroblast (CAFs) and mesenchymal stromal cells. It has been known for many years CAFs promote PCa when combined with benign prostate epithelial cells in mouse model studies and are required for the transformation of prostate epithelial cells and tissues [8]. Additionally, fibroblasts are considered non-professional innate immune cells (reviewed in [9]). They play an important role in innate and adaptive immunity, particularly in the early innate immune response, due to their ability to produce high levels of type I IFN [10]. Together these stromal components form a complex network that significantly influences tumor behavior and response to therapy (reviewed in [6,7]).

In PCa, the degree of DNA methylation correlates with the expression of cancer-associated genes (such as androgen receptor signaling) and identifies cancer stages [11]. DNA methylation of CpG sites is associated with an increased risk of metastasis in untreated men with localized PCa [12].

The interactions between tumor and stromal cells can trigger epigenetic alterations in tumor-adjacent stroma, particularly in fibroblast [13]. These interactions can lead to gene expression changes, alterations in cellular phenotype [14], and transforming of normal fibroblast into carcinoma-associated fibroblast [15]. Stromal epigenetic changes can also alter metabolic and neuroendocrine reprogramming in prostate cancer [16]. Stromal methylation changes can impact the response to treatment through altering drug efficacy within the tumor microenvironment, modulating the immune response to the tumor and effectiveness of immunotherapy [17].

Emerging studies suggest that the stroma exhibits distinct methylome characteristics in aggressive prostate cancer compared to the moderate form [15]. However, to the best of our knowledge, no studies directly compared stromal DNA methylation patterns in PCa patients of genetic ancestries. Consequently, it remains largely unknown how aberrant DNA methylation impacts the prostate tumor microenvironment in AA and EA men.

In prior studies, using transcriptome analysis, we found that the TAS of AA had many down-regulated genes relative to EA. Notably, many of these down-regulated genes encoded proteins associated with immune response functions [18,19]. In this study, we employed a genome-wide methylation sequencing approach to determine the differences in DNA methylation patterns within the tumor microenvironment of AA and EA PCa patients. Our results reveal strikingly higher global and gene-specific DNA methylation in the stromal component of AA compared to EA patients. These findings may shed light on the potential role of stromal DNA methylation in contributing to the observed disparities in PCa outcomes between these populations.

## 2. Materials and Methods

### 2.1. Patient Data

Prostate cancer tissues were collected using IRB-approved protocols at the Baylor College of Medicine (BCM), the Medical University of South Carolina (MUSC), and the University of California, Irvine (UCI). Clinical data were provided to study members as de-identified data elements (Supplementary Dataset S1, ST1A).

### 2.2. Sample Preparation and DNA Methylation Enrichment

For each patient, a pathologist marked a hematoxylin/eosin-stained (H&E) slide to identify tumor-adjacent stroma (TAS) (Supplementary Figure S1A). Multiple matching 20-micron-thick tissue sections were mounted on plastic microscope slides. The sites of the tumor and TAS were identified by superimposing each plastic slide on the marked H&E slide. TAS tissue was removed from the plastic slide with a 2 mm-diameter punch biopsy needle.

Genomic DNA (gDNA) for each sample was isolated from five punches and retrieved by deparaffinization (Qiagen, Venlo, Netherlands, Cat #19093) and purification (Norgen kit, Thorold, ON, CA, Cat. #54300). The concentration, yield, and purity of DNA were assessed by Qubit dsDNA Broad Range. Fragmented formalin-fixed paraffin-embedded DNA was sonicated to produce DNA fragments between 200 and 350 bp. Fragmented gDNA was then used to capture methylated DNAs (MethylCollectorTM Ultra, Active Motif, Carlsbad, CA, USA, Cat #55005). Methylated fractions were then used to construct standard fragment libraries (Bioo Scientific’s NEXTFLEX Rapid DNA-Seq Kit 2.0, Austin, TX, USA). For sequencing, all DNA sizes were selected using sample purification beads and sequenced at the UCI high-throughput genomic core facility.

### 2.3. Pathway and Network Analysis

Molecular canonical pathways and network analyses of statistically significantly differentially methylated genes of AA TAS (*n* = 17) compared to EA TAS (*n* = 15) (*p*-value < 0.05) were determined using Qiagen Ingenuity Pathway analysis (IPA).

### 2.4. Global DNA Methylation Analysis of AA and EA TAS Using Long Interspersed Nuclear Element-1 (LINE-1)

Global DNA methylation of AA TAS (*n* = 12) and EA TAS (*n* = 8) was measured using the LINE-1 assay kit (Active Motif, Carlsbad, CA, USA, Cat #55017). Briefly, genomic DNA (gDNA) was digested using the restriction enzyme Mse1 to generate appropriately sized fragments (290–300 bp) to hybridize biotinylated consensus sequences (probes) corresponding to human LINE-1 transposons. The 290 bp Mse1 fragments hybridize to LINE-1 probes, which contain 10 detectable CpG sites out of 76 cytosine. Hybridized samples were transferred and immobilized to 96-well streptavidin-coated plates, and methylated CpG sites were identified using a 5-methylcytosine antibody, HRP conjugate secondary antibody, and colorimetric detection reagents (optical density, OD450).

### 2.5. Carcinoma-Associated Fibroblast (CAF) Tissue Culture

The total number of culture preparations is summarized in Supplementary Dataset S1. A thin slice of tissue from the received biopsy punch was cut and fixed in 10% neutral buffered formalin, then embedded in paraffin for H&E staining, and regions containing the tumor were confirmed by a pathologist. The rest of the tissues were cut into 1–2 mm^3^ pieces for digestion using sterile techniques. To ensure that tissue strips contain tumor-adjacent stroma largely free of other components, one face of the strip was removed with a scalpel and used for frozen section preparation. If further trimming was necessary, the tumor–stroma boundary was marked on the back of the frozen section slide under a microscope using a Pilot Ultrafine permanent fine point pen. The top of the slide was sterilized with an alcohol swab and dried, and the tissue strip was overlaid on the corresponding frozen section for manual trimming in the biosafety cabinet. Trimmed tissue was digested by collagenase type I and hyaluronidase type II at 37 °C in the cell culture incubator, as described [8]. Cell suspensions were cultured in stromal cell selection media consisting of RPMI medium supplemented with 5% FCS, 1 nM testosterone, and 10 ng/mL fibroblast growth factors, which selected for fibroblasts over succeeding passages (Supplementary Figure S1B,C,D). Cancer-associated fibroblasts (4 AA and 4 EA) were cultured for 6 passages and then treated with 5 µM 5-Azacytidine (5-AzaC) or vehicle/control for 24 h. Following treatment, CAFs were cultured for 10 days in a drug-free medium, with media changed every 2–3 days. No visible changes were observed in drug-treated cells compared to vehicle-treated cells. Genomic DNA was isolated using commercial reagents (Norgen Biotek, Cells and Tissue DNA Isolation Kit, Thorold, ON, CA, Cat #53100). Methylation enrichment and sequencing methods, similar to those applied to tumor-adjacent stroma from formalin-fixed paraffin-embedded (FFPE) tissues of PCa patients, were used to analyze CAFs at three time points: untreated (d0), 1-day treatment (d1), and 10 days post (d10) 5-AzaC treatment. All primary cultured CAFs were tested for mycoplasma contamination using PCR and confirmed to be free of mycoplasma.

### 2.6. DNA Methylation Analysis

MBD-sequencing reads from AA TAS (*n* = 17) and EA TAS (*n* = 15) were aligned to the human genome reference build 38 (GRCh38) and deduplicated using Strand NGS as previously described [19,20]. Reads from AA CAFs (*n* = 3) and EA CAFs (*n* = 4) at three time points (d0, d1, and d10) following 5-AzaC treatment were aligned and deduplicated using the same methodology. DNA methylation enrichment was determined using a model-based analysis of ChIP-Seq (MACS) in Strand NGS 4.1 [21]. The parameters provided to the Strand NGS implementation of MACS included an average fragment length of 300 bp, effective genome size of 2.7 × 10^8^ bp, enrichment factor limit of 5 (lower) and 50 (upper), a *p*-value < 0.05, and no control sample. In the absence of a control sample, a dynamic lambda parameter was estimated for each MBD-seq sample for each read-enriched region. This was accomplished by determining the maximum read distribution (modeled by a Poisson distribution) across three windows: the whole genome, within 5000 bp, and within 10,000 bp centered around the peak. The fold enrichment factor was quantified as the ratio of peak height to the dynamic lambda parameter, and a corresponding *p*-value was generated for each enriched peak. Enriched methylated regions (i.e., peak sets) were subjected to further analysis using the R package DiffBind version 3.19.0 [22]. Briefly, consensus peaks were identified by locating regions with high levels of methyl enrichment proportional to their background and comparing those regions across all samples. If a region contained methyl enrichment in a minimum of 2 samples, that region was considered a consensus peak for downstream analysis. A summit was calculated from this consensus peak set as the location containing the highest degree of read overlap. To identify the region of true enrichment, the region containing methyl enrichment was trimmed and recentered by including 250 bp around the summit for a total region size of 501 bp. A read count matrix was generated for all samples using the summit peaks as new regions. Differential methylation analysis was performed using DESeq2, in combination with its normalization using default parameters, to identify statistically significant differentially methylated regions (DMRs) in AA TAS (*n* = 17) vs EA TAS (*n* = 15). DMRs with *p*-value < 0.05 were selected, and genes overlapping with the methylated region were identified using the NCBI GRCh38.p14 genome assembly. Global DNA methylation patterns were investigated between AA and EA TAS by comparing the average methylation in each cohort across all genomic regions (Mann–Whitney U test, *p*-value < 0.05). Heatmaps were generated with the heatmap R package version 1.32.0 and clustered using Euclidean distances with complete linkage.

### 2.7. Tumor Suppressor Gene Analysis

We used the tumor suppressor gene database (TSGene 2.0) [23] to detect tumor suppressor genes (TSG) across multiple cohorts. This database contains 1217 human TSGs containing 1018 protein-coding and 199 non-coding genes. We identified statistically significantly differentially methylated genes (*p*-value < 0.05) in AA TAS vs. EA TAS and cross-referenced with the TSGene database.

### 2.8. Global DNA Methylation Analysis Using LINE-1 Assay

To analyze global DNA methylation using a LINE-1 assay, a standard curve was generated using 7 DNA standards containing known levels of LINE-1 methylation included in the kit (Active Motif, Carlsbad, CA, USA, Cat #55017). The methylated and non-methylated DNA standards, derived from human Jurkat genomic DNA at 10 ng/µL, were prepared using the manufacturer’s recommendations. The methylated standard was treated with M.SssI and fragmented using MseI, while the non-methylated standard was amplified to remove endogenous methylation. DNA standards with known methylation levels (0–100 ng) were performed in triplicate. The analysis was performed by averaging the optical density (OD) 450 values for the triplicate blanks and standards. We calculated the %5-mC relative to the total of cytosine content per manufacturer direction. The total cytosine content is determined to be in the range of 1% for 10 ng and 13% for 100 ng methylated LINE-1. We used the curve fitting tool in GraphPad to plot the %5-mC associated with each standard along the x-axis and the averaged OD values along the y-axes. For the patient samples, we used 50–100 ng Mse1 fragmented DNA per well, and the experiment was conducted in duplicates. Since the 100 ng standard curve was used to extrapolate %5-mC for patient samples, the extrapolated %5-mC values for DNA samples with less than 100 ng (*n* = 2) are divided by the DNA amount and multiplied by 100% to estimate the relative level of methylated DNA in samples with <100 ng.

MBD-seq data for 32 TAS (AA *n* = 17, EA *n* = 15) and 7 CAF (AA *n* = 3, EA *n* = 4) samples at different time points before, after, and post 5-AzaC treatment (d0, d1, and d10) have been deposited in the GEO database (GSE261520).

## 3. Results

### 3.1. DNA Methylation Profiles of Tumor-Adjacent Stroma in African American and European American Prostate Cancer Patients

**Figure 1:**
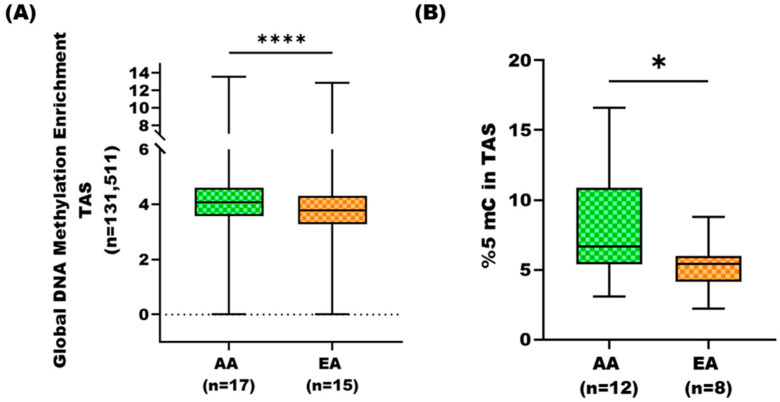
Global DNA methylation analysis in tumor-adjacent stroma of African American and European American prostate cancer patients. (**A**) Box plot comparisons of global DNA methylation based on MBD-seq data in TAS of AA (*n* = 17) and EA (*n* = 15) PCa patients. The average methylation enrichment across 131511 regions was compared between cohorts. AA patients exhibited significantly greater global DNA methylation (*p*-value < 0.001, Mann–Whitney U test). (**B**) Box plot comparison of global %5-methylcytosine (%5-mC) content in AA and EA TAS using LINE-1 assay. AA TAS exhibited significantly higher levels of 5-methylcytosine (*p*-value = 0.039, Mann–Whitney U test). * = *p*-value < 0.05 and **** = *p*-value < 0.0001.

To investigate DNA methylation variations in TAS among PCa patients with different geographical ancestries, we employed MBD-seq to quantify methylated regions in tumor-adjacent stroma of AA and EA cohorts (Supplementary Dataset S1, ST1A). We identified methylated regions, using model-based analysis of ChIP-Seq (MACS) peak calling [21] and DiffBind [22]. MACS is traditionally used for detecting transcriptional factor binding sites and histone modification-enriched regions with broad peaks, and it can also be applied to methylation enrichment-based sequencing (i.e., MBD-seq) data [21]. Global DNA methylation was determined, normalized, and averaged for each methylated region (*n* = 131511) across samples for each group (i.e., 17 AA and 15 EA). Comparison using the Mann–Whitney U test revealed that the AA TAS had strikingly increased DNA methylation at global levels as compared to the TAS of EA patients (*p*-value < 0.0001) (Figure 1A, Supplementary Dataset S2, ST2A).

### 3.2. Global DNA Methylation Analysis of AA TAS and EA TAS Using Long Interspersed Nucleotide Elements Assay

To independently verify the global DNA methylation differences between AA and EA tumor-adjacent stroma, we employed LINE-1 assay. LINE-1 elements, comprising 17% of the human genome, serve as surrogate markers for global DNA methylation patterns [24]. We analyzed TAS samples from an-independent cohorts of AA (*n* = 12) and EA (*n* = 8) PCa patients not included in the MBD-seq analysis (Supplementary Dataset S1, ST1A). The global methylation level (i.e., %5-mC) was determined for each sample and compared between the two cohorts. Consistent with our MBD-seq results, we observed significantly higher levels of %5-mC in AA TAS versus EA TAS (*p*-value < 0.03) (Figure 1B).

### 3.3. Differentially Methylated Regions/Genes in the Tumor-Adjacent Stroma of AA vs. EA PCa Patients

**Figure 2:**
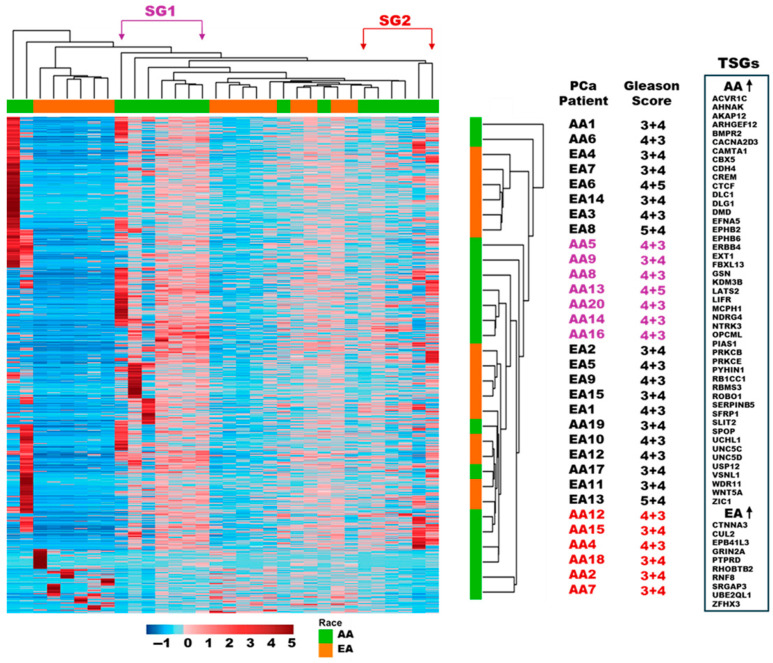
Hierarchical clustering analysis of DNA methylation in tumor-adjacent stroma. The dendrogram on the top and right lists the patient samples from AA (*n* = 17) and EA (*n* = 15) cohorts. This illustrates the relatedness of DNA methylation across 1659 statistically significantly differentially methylated regions (*p*-value < 0.05) that correspond to 1557 protein-coding genes for each patient. Alongside the dendrogram (rotated 90° and enlarged on the right), patients’ ancestry and Gleason score are depicted. On the right side, the tumor suppressor activity of significantly differentially methylated genes is displayed. The list indicates which genes are more methylated in AA and which ones are more methylated in EA PCa patients. The level of DNA methylation is depicted as a color intensity on a z-score scale; (TAS) tumor-adjacent stroma, (AA) African Americans, (EA) European Americans, (SG1) subgroup 1, (SG2) Subgroup 2, and (PCa) prostate cancer.

Our analysis revealed the identity of 3268 statistically significant differentially methylated regions (DMRs, *p*-value < 0.05) in AA TAS (*n* = 17) compared to EA TAS (*n* = 15) (Supplementary Dataset S2, ST2B). Of these DMRs, 1977 (60%) were associated with 1885 genes (1557 protein-coding genes and 328 pseudogenes), while the remaining DMRs were unspecified loci (*n* = 1291, 40%). Of the 1557 protein genes, 1379 (89%) showed increased DNA methylation in AA TAS when compared to the EA TAS cohort (Figure 2). These included immune-related genes such CD28, GZMB, CD226, and IL-7.

We identified 91 genes with tumor-suppressive properties that exhibited significantly increased DNA methylation in AA TAS compared to EA TAS (*p*-value < 0.05). Of the 91 genes, 47 have been previously reported in PCa [23] including BMPR2, ERBB4, ROBO1, SFRP1, EXT1, EPHB2, WNT5A, FBXL13, and SPOP (Figure 2, Supplementary Dataset S2, ST2B).

Conversely, 178 protein-coding genes (11%) showed increased DNA methylation in EA TAS compared to AA TAS. Among these were genes with tumor-suppressive activities such as CUL2, CTNNA3, and RNF8 (Figure 2, Supplementary Dataset S2, ST2B).

### 3.4. Subgroup Analysis of DNA Methylation Patterns in AA TAS

**Figure 3:**
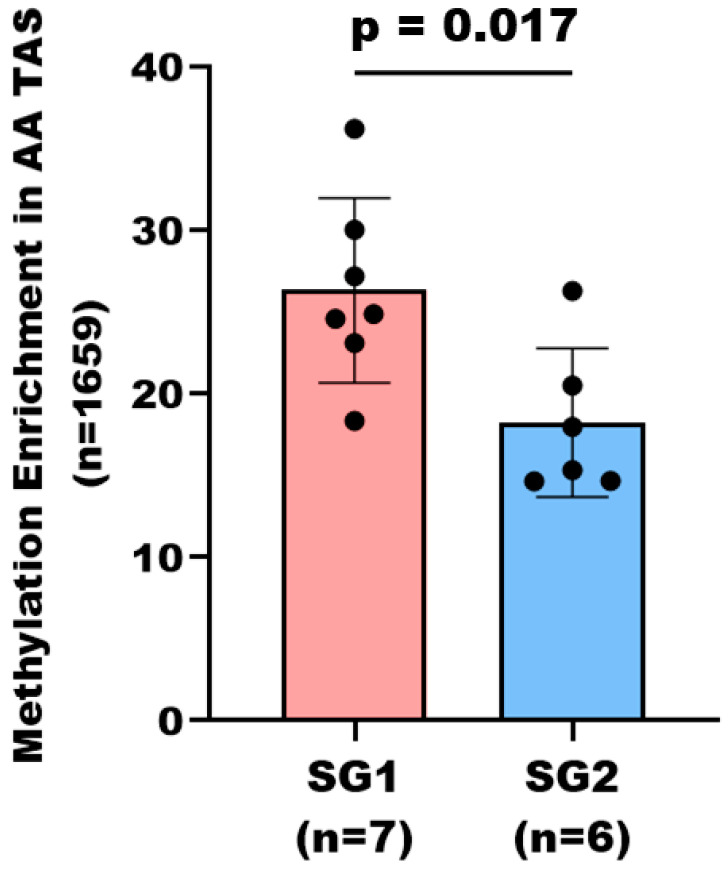
Subcategorization of AA PCa patients is based on stromal DNA methylation levels. The bar graph depicts average methylation enrichment in SG1 (*n* = 7) and SG2 (*n* = 6) of AA TAS samples. Analysis covers 1659 statistically significant differentially methylated regions corresponding to 1557 protein-coding genes. Methylation enrichment was significantly different between the two subgroups (*p*-value < 0.05, Mann–Whitney U test). Each dot represents an individual patient within the subgroup. Of the seven patients in SG1 with greater DNA methylation, six patients had a Gleason score of 4 + 3 or higher. In contrast, two out of six patients in SG2 had a Gleason score of 4 + 3 or higher.

Hierarchical clustering analysis of the statistically significant DMRs of TAS (*n* = 1659, corresponding to 1557 protein-coding genes, *p*-value < 0.05) revealed two distinct subgroups within the AA patients based on their DNA methylation levels; subgroup 1 (SG1) with considerable DNA methylation, and subgroup 2 (SG2) with less DNA methylation (Figure 2, Supplementary Dataset S2, ST2B). Analysis of Gleason scores within the subgroups revealed that 86% (6/7) of patients in SG1 had higher Gleason scores (4 + 3 and 4 + 5), compared to only 33% (2/6) of patients in SG2 with a Gleason score greater than 3 + 4. This distribution suggests that SG1, with more extensive DNA methylation, predominately consists of patients with more advanced disease (Figure 3, Supplementary Dataset S2, ST2B).

### 3.5. Pathway Analysis of Differentially Methylated Genes in TAS of AA and EA Patients

**Figure 4:**
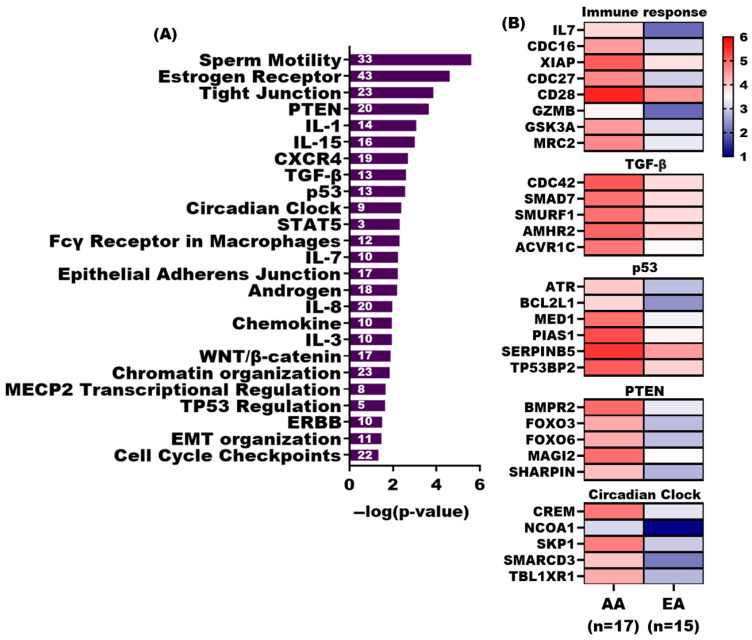
Pathway analysis of significantly methylated genes in AA TAS vs EA TAS. (**A**) Bar diagrams depicting the selected significant canonical pathways (*p*-value < 0.05) associated with significant differentially methylated genes showing increased DNA methylation in AA TAS (*n* = 1379) compared to EA TAS. Pathway analysis was conducted using QIAGEN Ingenuity Pathway Analysis. (**B**) Heatmap displaying DNA methylation enrichment of selected genes in significant pathways (*p*-value < 0.05) with increased DNA methylation in AA TAS (*n* = 17). DNA methylation enrichment values for AA TAS (*n* = 17) and EA TAS (*n* = 17) were transformed Log2.

Our previous studies have shown that the tumor-adjacent stroma of AA had numerous down-regulated genes relative to EA PCa patients, many of which play key roles in immune responses [18,19]. Pathway analysis of genes with significantly more DNA methylation in AA TAS (*n* = 1379) revealed significant association (*p*-value < 0.05) with several immune response pathways, including IL-15, IL-8, IL-1, IL-7, and IL-3 signaling as well as a STAT5 pathway. Other affected pathways were PTEN, p53, circadian clock regulation, cell cycle check points, TGF-β signaling, Wnt/β-catenin signaling, androgen signaling, ERBB, and sperm motility (Figure 4A,B, Supplementary Dataset S2, ST2C).

Among the significant pathways (*p*-value < 0.05) associated with genes with significantly more DNA methylation in EA TAS (*n* = 178) are DNA double-strand break response, ATM signaling, the role of CHK proteins in cell cycle checkpoint control, and Toll-like receptor signaling (Supplementary Dataset S2, ST2E).

### 3.6. Network Analysis of Differentially Methylated Genes in AA and EA TAS

**Figure 5:**
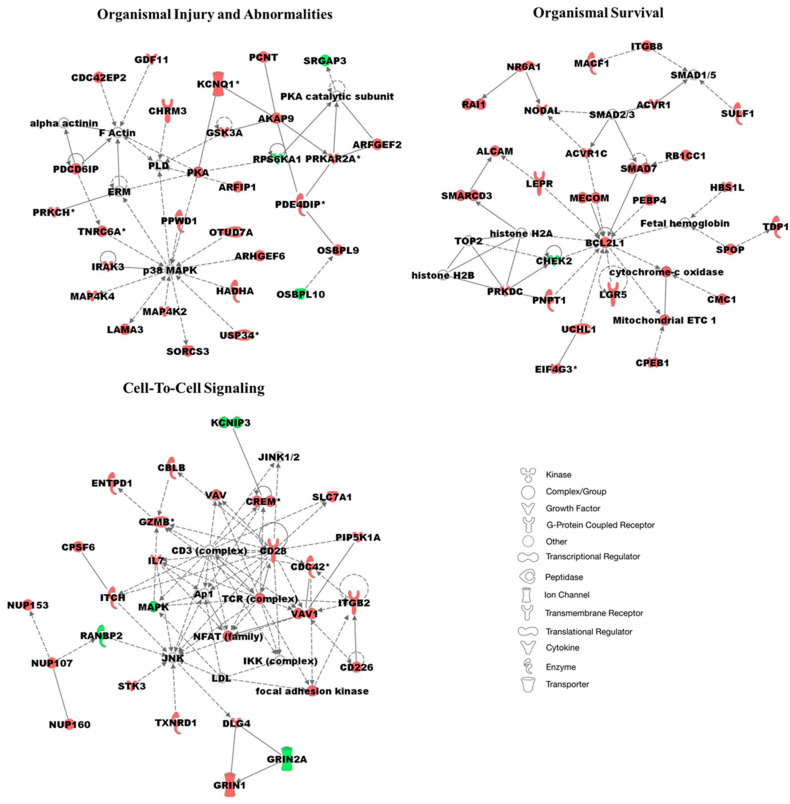
Top three networks associated with significantly differentially methylated genes in TAS of AA vs. EA (i.e., 1557 protein-coding genes). Node shapes are defined in the key to the right of the networks. Red color nodes indicate genes of the network that are more methylated in AA TAS vs. EA TAS. Darker shades of the nodes indicate higher methylation values. Green color nodes represent genes with decreased DNA methylation in AA TAS compared to EA TAS. Solid lines represent direct interactions, while dotted lines represent indirect interactions. White colored nodes are in the network, but not in our dataset. Nodes marked with an asterisk (*) indicate that multiple identifiers in the dataset map to a single gene.

We performed network analysis on 1557 statistically significant differentially methylated genes (*p*-value < 0.05) in AA TAS and EA TAS. This analysis revealed multiple networks, including those involved in organismal injury and abnormalities, survival, and cell-to-cell signaling and interactions (Figure 5, Supplementary Dataset S2, ST2D). Several major hub genes were identified as either more methylated or associated with other methylated genes in AA patients, including p38 MAPK, a gene associated with inflammation and cell differentiation. BCL2L1 emerged as a central hub gene with increased DNA methylation in AA and TAS. This gene directly interacts with SMAD7, an immune response and growth factor gene, and part of the PTEN signaling pathway. CD28, a key immune response hub gene, and granzyme B (GZMB), which directly interacts with CD28, were both found to be more methylated in AA TAS (Figure 5, Supplementary Dataset S2, ST2D).

### 3.7. Development and Characterization of Carcinoma-Associated Fibroblasts (CAFs) from Primary Prostate Tumors

**Figure 6:**
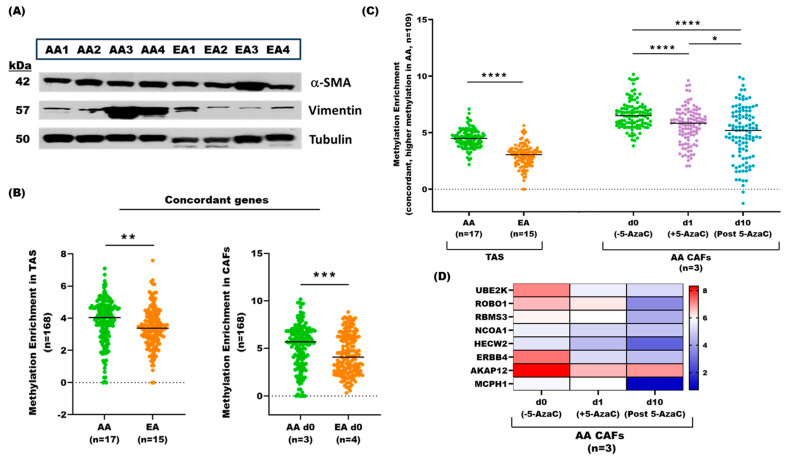
Methylome analysis of patient-derived tumor-adjacent CAF cultures. (**A**) Western blot analysis of α SMA and vimentin (fibroblast markers) and tubulin (housekeeping gene) in AA (*n* = 4) and EA (*n* = 4) CAF primary cultures at passage 6. (The original and uncropped Western images with band quantification are included in the supplementary material file (Supplementary Figure S1C,D). (**B**) Concordance analysis of statistically significantly differentially methylated genes in AA vs. EA TAS (*n* = 1885) and AA vs. EA CAFs (*n* = 3255). Box plots show DNA methylation distribution of 168 concordant methylated genes in AA TAS (*n* = 17) vs. EA TAS (*n* = 15) (left panel) and AA CAFs (*n* = 3) vs EA CAFs (*n* = 4) (right panel). Average DNA methylation levels differ significantly between AA and EA in both TAS and CAFs (*p*-value < 0.001 and < 0.01, respectively, according to the Mann–Whitney U test. (**C**) Methylation status of 109 genes with higher methylation in both AA CAFs and AA TAS at three time points: day 0 (d0; untreated), day 1 (d1; 5-AzaC treatment), and day 10 (d10; post 5-AzaC treatment). Average DNA methylation in AA CAFs (*n* = 3) decreased significantly at d1 (*p*-value < 0.0001, Mann–Whitney U test) and further decreased in post d10 (*p*-value < 0.0001, Mann–Whitney U test) compared to d0. (**D**) Heatmap depicting average DNA methylation enrichment of key selected concordant genes in AA CAFs (*n* = 3) across three time points (d0, d1, and d10). * = *p*-value < 0.05, ** = *p*-value < 0.01, *** = *p*-value < 0.001, and **** = *p*-value < 0.0001. Note: one AA CAF is excluded from the analysis due to insufficient sequencing reads.

We established primary cell cultures of tumor-adjacent stroma (Supplementary Figure S1A,B) from fresh prostatectomy tissues of AA (*n* = 4) and EA (*n* = 4) patients to investigate their suitability as a model for exploring mechanistic and phenotypic effects observed in tumor-adjacent stroma from FFPE PCa tissues. We carried out Western blot analyses of the representative CAF lines (all at passage 6) for classical markers of prostate myofibroblasts of TAS (i.e., α-smooth muscle actin and vimentin), and found strong expressions in all cases (Figure 6A), confirming the myofibroblast nature of our CAFs.

To evaluate the differences in DNA methylation between AA and EA cohorts and the effect of a demethylating agent on CAFs, we used the nucleoside analog 5-Azacytidine (5-AzaC), which is currently used in treating hematological malignancies. CAFs from both cohorts were treated with 5 µM 5-AzaC for 1 day, followed by 10-day culture in a drug-free medium. We applied the same analysis method used for TAS from FFPE tissues. One AA CAF primary line was excluded from the analysis due to insufficient sequencing reads.

### 3.8. Comparative Analysis of DNA Methylation Patterns Between Carcinoma-Associated Fibroblast Derived from Primary Prostate Tumors and Tumor-Adjacent Stroma from PCa Patients

We compared statistically significant differentially methylated genes, including both protein-coding genes and pseudogenes in untreated AA CAFs (*n* = 3) vs untreated EA CAFs (*n* = 4) (*n* = 3255, *p*-value < 0.05, Supplementary Dataset S3, ST3A) with those identified in AA TAS (*n* = 17) vs. EA TAS (*n* = 15) from FFPE tissues (*n* = 1885, *p*-value < 0.05).

Of the 439 overlapping genes between TAS and CAFs, 168 were concordantly methylated in both datasets (Figure 6B, Supplementary Dataset S3, ST3B). Of 168 genes that overlapped between the TAS and CAFs, 109 exhibited increased and 59 decreased DNA methylation in AA compared to EA patients. We focused on 109 genes with higher DNA methylation in both AA CAFs and AA TAS and found a significant decrease in average DNA methylation after 1 day of 5-AzaC treatment compared to untreated CAFs (day 0) (*n* = 3; *p*-value < 0.000, Mann–Whitney U test). Our data reveal a further significant decrease in average DNA methylation in AA CAFs (*n* = 3) 10 days post-5-AzaC treatment compared to both untreated and 1-day-treated samples (*n* = 3; *p*-value < 0.0001, Mann–Whitney U test) (Figure 6C, Supplementary Dataset S3, ST3C). These results suggest progressive demethylation effects of 5-AzaC treatment on identified genes in AA CAFs.

Notably, genes that exhibited reduced methylation following 1 day of 5-AzaC treatment and showed further demethylation 10 days post treatment included ERBB4, EVI5, UBE2K, ROBO1, RBMS3, AKAP12, PIAS2, and LAMB1 (Figure 6C,D, Supplementary Dataset S3, ST3C). In contrast, genes with more DNA methylation in both EA CAFs and EA TAS (*n* = 56) showed no statistically significant decrease in average DNA methylation after and post treatments.

## 4. Discussion

The mechanism of how ancestry contributes to aggressive prostate cancer in African American patients remains incompletely understood. While there is evidence for genetic polymorphisms, differences in gene expression, and differential DNA methylation between EA and AA PCa [18,19], our study highlights the critical role of the tumor microenvironment in prostate cancer disparity.

Recent advances in cancer genetics and epigenetics emphasized the importance of the tumor microenvironment [25]. Tumor-associated stroma (TAS), including reactive stroma, plays a critical role in tumorigenesis of PCa [26,27]. TAS not only produces stimulatory paracrine growth factors that act on epithelial cells, but also profoundly affects immune cells through secretions as well as with direct interactions [28]. Our previous work demonstrated the diagnostic value of studying TAS compared to normal stroma in PCa patients at the RNA level [29,30]. The current study builds upon these findings by exploring epigenetic differences in TAS between AA and EA patients.

The role of epigenetic alterations in PCa progression gained increasing attention in recent years. Genome-wide DNA methylation of the stromal component of the tumor microenvironment is associated with aggressive disease in PCa patients [12]. While previous studies identified differences in methylome between AA and EA tumor, the epigenetic landscape of tumor-adjacent stroma in these populations remained largely unexplored.

Our analysis revealed several key findings. First, we found strikingly higher global DNA methylation in TAS of AA than in EA patients (*p*-value < 0.05). This finding was further supported by increased DNA methylation of LINE-1 transposable elements in AA TAS (*p*-value < 0.039). The LINE-1 retrotransposon component serves as a surrogate marker for global genomic DNA methylation [31]. While previous studies reported global hypomethylation of LINE-1 in cancer cells compared to normal cells [32], our study is novel in its focus on DNA methylation in the tumor microenvironment of patients of different ancestral backgrounds.

Recent studies highlighted the beneficial role of the demethylation of retrotransposable elements, including endogenous retroviruses and LINE-1, in cancer. Demethylation and de-repression of retrotransposons can lead to the formation of immunostimulatory dsRNA, activating anti-tumor immune response genes [33]. The higher global DNA methylation observed in AA TAS may potentially suppress these beneficial immune responses. Furthermore, the observed differences in global DNA methylation in the tumor microenvironment may affect how AA patients respond to epigenetic-modifying drugs, such as the demethylating agent 5-Azacytidine (5-AzaC). This finding may suggest the need for personalized approaches to epigenetic therapies in PCa treatment.

Our genome-wide DNA methylation analysis in TAS of AA and TAS of EA PCa patients revealed significant differences in gene-specific methylation patterns. We identified 1557 protein-coding genes that were significantly differentially methylated in AA as compared to EA, the majority, 1379 (89%) of which exhibited increased methylation in AA (*p*-value < 0.05). Several important genes with significantly higher DNA methylation in TAS of AA emerged, including the immune response genes CD28, CD226, GZMB, and IL-7. These genes play important roles in activating CD4+T, CD8+T, natural killer, immature, and mature antigen-presenting cells such as dendritic cells. The hypermethylation of these key immune genes in AA TAS may have a significant implication for anti-tumor immunity. For instance, the co-immunostimulatory genes CD226 and CD28 are essential for T cell activation and enhancement of anti-tumor immune responses [34,35]. Their hypermethylation could potentially suppress T-cell-mediated anti-tumor activity in AA patients. GZMB, which induces apoptosis and modulates the immune response in the tumor microenvironment, has been associated with improved clinical outcome and overall survival when its methylation is lower [36]. Additionally, IL-17 demonstrated anti-tumor activity and therapeutic potential in PCa [37]. Hypermethylation of this gene in AA TAS could potentially diminish its beneficial effects.

We also identified a significant association between genes with more DNA methylation in AA TAS vs. EA TAS and immune response pathways, including IL-8, WNT/β-catenin, STAT5, chemokine, IL-15, and IL-3. Alterations in the IL-8 signaling pathway in the tumor microenvironment have been shown to enhance the aggressiveness of PCa [38]. Similarly, the WNT/β-catenin signaling pathway blocks CD8+T cells from entering tumor sites and prevents dendritic cells from activating an immune response, which helps tumors evade the immune system and decreases the success of treatments such as immune checkpoint inhibitors [39].

Our prior transcriptome studies, utilizing both frozen and FFPE tissues, revealed significant down-regulation of numerous genes in the tumor-adjacent stroma of AA vs. EA patients. These down-regulated genes were involved in several immune-related pathways, including natural killer cell signaling, B-cell receptor signaling, dendritic cell maturation, the antigen presentation pathways, and mTOR signaling [18,19]. The convergence of our current methylation data with these prior expression findings strongly suggest immune dysfunction in the tumor microenvironment as a potential key factor for PCa disparities between the two groups.

Studies of over 1900 patients with metastatic castration-resistant PCa revealed a significant survival benefit in favor of African American patients who received Sipuleucel-T immunotherapy in comparison to European Americans [40]. This differential response suggests potential genetic, epigenetic, and cellular immune differences between AA and EA patients, which may influence the treatment outcomes.

Among other methylated pathways in AA TAS was the circadian clock regulation. While alterations in the regulation of circadian clock machinery, which acts as a tumor suppressor, have been reported in tumor cells of AA patients [41], our findings highlight the involvement of the tumor microenvironment, TAS, in these processes.

We found increased DNA methylation in AA TAS compared to EA TAS for several known tumor suppressor genes, including SFRP1 and SPOP. This finding is particularly significant for several reasons. DNA methylation and silencing of SFRP1 disrupts the Wnt signaling pathway, which is aberrantly activated in PCa [42]. The hypermethylation of SFRP could potentially contribute to enhanced Wnt signaling, particularly in the tumor microenvironment, promoting disease progression. The SPOP gene is frequently mutated in approximately 10% of PCa patients and is associated with increased global DNA methylation [43]. To our knowledge, this is the first study to report increased DNA methylation of tumor suppressor genes in the TAS of PCa patients across different genetic ancestries. Stromal reprogramming of DNA methylation could contribute to a more permissive stromal microenvironment for tumor growth, potentially explaining, at least in part, the more aggressive PCa outcomes often observed in AA patients.

Another key finding of this study is the heterogeneity of DNA methylation patterns within the TAS of AA PCa patients. DNA methylation stratified AA patients into two potentially distinct subgroups. Subcategorization of AA PCa patients based on the DNA methylation profile of TAS has not been previously described. Importantly, the DNA methylation differences within the AA patient groups correlate with their Gleason score. The AA subgroup with higher stromal DNA methylation was associated with more advanced disease (i.e., Gleason scores 4 + 3 and 4 + 5). This observation suggests, at least in part, that DNA methylation patterns in AA TAS may not be homogeneous, and opens the possibility for a more personalized approach to prognosis and treatment. Patients in the high-methylation subgroup might benefit from targeted epigenetic therapies.

Our comparative analysis of tumor-adjacent stroma from FFPE PCa tissues and primary cultured carcinoma-associated fibroblast identified 109 overlapping genes with higher methylation in both AA TAS and AA CAFs compared to their EA counterparts (*p*-value < 0.001). Among these concordantly methylated genes, several have known tumor-suppressive activities in PCa, including ERBB4, MCPH1, RBMS3, and AKAP12, High DNA methylation levels of ERBB4 have been associated with poor prognosis and aggressive disease in breast cancer patients [44]. Similarly, deletion of the DNA damage response gene (i.e., MCPH1) is linked with aggressive disease and shorter survival in multiple cancers, including prostate cancer [45]. In PCa, the tumor suppressor gene RBM3 inhibits the stemness remodeling of PCa cells by osteoblasts, thereby preventing metastasis [46]. Aberrant DNA methylation of A kinase anchor protein 12 (AKAP12) has been reported in PCa patients with a high Gleason score [47]. Interestingly, we observed decreased DNA methylation in these genes 10 days post-5-AzaC treatment in AA CAFs compared to both untreated (d0) and 1-day (d1)-treated cells. Studies suggest that following post-5-AzaC treatment, some genes can remain stably demethylated for several months [48]. This may indicate the potential for long-lasting epigenetic reprogramming of the stromal cells.

5-AzaC, a nucleoside analog and demethylating agent, is a promising drug for targeting stromal epigenetics by inhibiting DNA methyltransferases (DNMTs). It effects both tumor cells and the stromal compartment. Studies have shown that combined inhibition of DNMT activities using 5-AzaC and JAK signaling, both in vitro and in vivo, results in long-term reversion of CAF-associated preinvasive activity and restoration of the wild-type fibroblast phenotype in lung, head, and neck carcinoma [49]. Additionally, 5-AzaC can modify the stromal component in pancreatic cancer, affecting CAF behavior and potentially restoring normal function [50]. Our observation of decreased DNA methylation in key genes following 5-AzaC treatment in AA CAFs may suggest a potential stromal reprogramming. Combination of 5-AzaC effects on stromal methylation and immune pathways activation may create a more favorable tumor microenvironment for anti-tumor responses. Furthermore, 5-AzaC exhibited antitumor efficacy in treatment of hematologic malignancies, including acute myeloid leukemia and myelodysplastic syndrome, while maintaining acceptable safety profiles [51].

While our study provides valuable insights into the epigenetic landscape of tumor microenvironment in AA and EA PCa patients, there are several limitations. We rely on self-identified races, which may be a low-precision proxy for genetic ancestry. MBD-seq excludes parts of the genome that are not methylated, limiting our ability to determine the precise genetic ancestry of each patient. DNA methylation patterns can be altered by lifestyles such as smoking, diet, exercise, and alcohol consumption. Moreover, tumor somatic mutation can disrupt normal methylation processes, leading to aberrant gene methylation [52]. These confounding variables should be considered in methylation studies. However, our datasets lack information on these parameters, which may contribute to the observed heterogeneity in DNA methylation between and within cohorts. Future large-scale studies should consider collecting and incorporating this data to provide a more comprehensive analysis of DNA methylation, particularly when comparing DNA methylation across different cohorts. The small sample size of primary cultured fibroblasts from patients of different races posed limitations on our ability to investigate the heterogeneity in DNA methylation within the AA CAFs, as we observed in our AA TAS cohort. Our study also highlights the complexity of the TAS, which comprises both an acellular and cellular component. While CAFs are a major significant part of the tumor microenvironment, the presence of diverse cell types adds complexity to the observed DNA methylation differences. Despite limitations, cultured CAFs remain suitable models for future research on the tumor microenvironment and to investigate the therapeutic effect of demethylation using epigenetic modulators in combination with standard of care therapy. Our study is among the first to examine stromal methylation in the context of race and prostate cancer, making it an important exploratory investigation. Our findings warrant and will guide future studies using larger patient cohorts to further explore the important relationship between race, stromal methylation, and prostate cancer outcomes.

## 5. Conclusions

Our study provides new insights into stromal methylation reprogramming that may contribute to the observed disparities in PCa outcomes between AA and EA patients. We demonstrated significant differences in stromal DNA methylation patterns between PCa patients of different genetic ancestries. African American PCa patients exhibited strikingly higher global methylation as well as gene-specific DNA methylation in tumor-adjacent stroma compared to their European American counterparts. Many immune response genes with higher DNA methylation in AA TAS were enriched in immune response pathways, suggesting a potential mechanism for altered immune response in the tumor microenvironment of AA patients. We observed intra-cohort heterogeneity in DNA methylation among AA patients, which underscores the complexity of PCa disparities. Comparative analysis identified many concordantly methylated genes in the tumor-adjacent stroma of PCa patients and cultured carcinoma-associated fibroblast derived from primary PCa patients, validating CAFs as a relevant cell model for studying stromal epigenetic alterations. We observed a decrease in average DNA methylation of concordantly methylated genes (those with more methylation in AA TAS and AA CAFs) in response to de methylating agent 5-AzaC treatments, suggesting the potential for epigenetic therapies in addressing stromal methylation alterations. In summary, this study provides a foundation for future studies into targeted epigenetic therapies and personalized approaches that consider the unique stromal methylation profiles of individual patients, particularly for AA patients who face disproportionally aggressive disease.

## Data Availability

MBD-seq data generated from this study (total of 53) is deposited in GEO Accession GSE261520 and will be available to the public on 15 September 2025. The other data sets used/generated in this study are available from the corresponding author upon reasonable request.

## Supplementary Materials

The following supporting information can be downloaded at: https://www.mdpi.com/article/10.3390/cancers17142399/s1, Supplementary Dataset S1: Clinical Data for African American (AA), European American (EA) tumor-adjacent stroma (TAS) and cancer-associated fibroblast (CAFs), Supplementary Dataset S2: Methyl Binding Domain sequencing analysis in AA tumor-adjacent stroma (*n* = 17) and EA tumor-adjacent stroma (*n* = 15), Supplementary Dataset S3: DNA Methylation Enrichment for AA and EA CAFs. Supplementary Figure S1: Example of selection of tumor-adjacent stroma and morphology of primary cultured CAF early in passage 5.

## Abbreviations

The following abbreviations are used in this manuscript:

5-AzaC	5-Azacytidine
AA	African American
CAFs	carcinoma-associated fibroblasts
DMRs	differentially methylated regions
d0	before treatment
d1	1 day after treatment
d10	10 days post-treatment
EA	European American
MACS	model-based analysis of ChIP-Seq
MBD-seq	methyl-binding domain sequencing
PCa	prostate cancer
SG1	subgroup 1
SG2	subgroup 2
TAS	tumor-adjacent stroma
TSGs	tumor suppressor genes
UCI	University of California Irvine
