# Supplementary material for: Genes Encoding Multiple Modulators of the Immune Response Are Methylated in the Prostate Tumor Microenvironment of African Americans
Source: Cancers (Basel). 2025 Jul 19;17(14):2399. doi: 10.3390/cancers17142399 (PMC12293404; doi:10.3390/cancers17142399)
Supplement: Supplementary file 1 [file cancers-17-02399-s001.zip › cancers-3685623-supplementary.pdf]

Supplementary Materials

Genes encoding multiple modulators of the immune response are methylated in the prostate tumor microenvironment of African Americans

Vinay Kumar [1,†], Tara Sinta Kartika Jennings [1,†], Lucas Ueta [1], James Nguyen [1], Liankun Song [2], Michael McClelland [3], Weiping Chu [3], Michael Lilly [4], Michael Ittmann [5], Patricia Castro [5], Arash Rezazadeh Kalebasty [6], Dan Mercola [1], Omid Yazdanpanah [6], Xiaolin Zi [2] and Farah Rahmatpanah [1,*]

Supplementary Dataset S1. Clinical Data for African American (AA), European American (EA) tumor-adjacent stroma (TAS) and cancer associated fibroblast (CAFs). Prostate cancer tissues were collected using IRB-approved protocols at Baylor College of Medicine (BCM), Medical University of South Carolina (MUSC), and University of California, Irvine (UCI). CAFs were prepared by tissue culture at UCI. Tumor stage and Gleason scores were determined by certified pathologists at MUSC, BCM and UCI.

Supplementary Dataset S1A: Clinical data for AA and EA TAS samples that underwent Methyl Binding Domain sequencing (MBD-seq) and Long Interspersed Nuclear Element-1 (LINE-1) analysis.

Supplementary Dataset S1B: Clinical data for AA and EA CAF that underwent MBD-sequencing and treatment with demethylation agent 5-Azacytidine (5-AzaC).

Supplementary Dataset S2. Methyl Binding Domain sequencing analysis in AA tumor adjacent stroma (*n* = 17) and EA tumor adjacent stroma (*n* = 15). MBD-sequencing reads from AA TAS (*n* = 17) and EA TAS (*n* = 15) were aligned to the hg38 genome reference and

deduplicated using Strand NGS. DNA methylation enrichment was computed and normalized using DESeq2. A consensus peak set was created by identifying all genomic regions that were methylated (based on Model- based Analysis of ChIP-seq (MACs) peak calling) across at least 2 samples (n = 32). Differentially methylated regions between AA and EA cohorts in the TAS were evaluated for each region (*p-value* < 0.05). Molecular canonical pathways and networks of statistically significantly differentially methylated genes of AA TAS (n = 17) compared to EA TAS (n = 15) was determined using Qiagen Ingenuity pathway analysis (IPA). The list of significant pathways is generated (*p-value* < 0.05).

Supplementary Dataset S2A: Global DNA methylation of tumor-adjacent stroma (TAS) of AA (n = 17) and EA (n = 15) PCa patients with methylation enrichment across 131511 regions.

Supplementary Dataset S2B: 3268 statistically significant differentially methylated regions (*p-value* < 0.05) in AA TAS *vs*. EA TAS PCa patients. Methylation enrichment data is provided for each AA and EA PCa patient.

Supplementary Dataset S2C: Canonical pathways associated with statistically significant differentially methylated genes. Analysis is based on 1379 genes with significantly higher DNA methylation in AA TAS compared to EA TAS. Significant canonical pathways (*p-value < 0.05*) associated with these genes listed.

Supplementary Dataset S2D: Networks of statistically significant differentially methylated genes (n = 1557) in AA vs EA TAS.

Supplementary Dataset S2E: Canonical pathways associated with genes with significantly higher (*p-value* < 0.05) levels of DNA methylation in EA TAS *vs.* AA TAS (n = 178). Significant canonical pathways (*p-value < 0.05*) associated with these genes listed.

Supplementary Dataset S3. DNA Methylation Enrichment for AA and EA CAFs. MBD-sequencing reads from AA CAFs (*n* = 3) and EA CAFs (*n* = 4) before and after 5-AzaC treatments (5 μM) were aligned to the hg38 genome reference and deduplicated using Strand NGS. DNA methylation enrichment factor was computed and normalized using DESeq2. A consensus peak set was derived from the aligned reads of AA and EA CAF samples, analyzed using the same method as TAS. The effect of 5-AzaC treatment on DNA methylation levels across different time points (ie., without 5-AzaC (d0), with 5-AzaC (d1), and 10 days post 5-AzaC (d10) listed. Statistically significantly differentially methylated TAS and CAF genes (*p-value* < 0.05) were selected for concordance in methylation direction for each cohort. Of the 439 overlapping genes between TAS and CAFs, 168 were concordantly methylated in both datasets. Methylated genes that had a concordant increase in methylation in AA TAS and CAFs (*n* = 109) were analyzed in AA CAFs for treatment with 5-AzaC.

Supplementary Dataset S3A: 5233 statistically significant differentially methylated regions corresponding to 3255 genes (*p-value* < 0.05) in untreated (d0) AA CAFs (*n* = 3) *vs.* untreated (d0) EA CAFs (*n* = 4). Methylation enrichment data provided for AA CAFs (*n* = 3) and EA CAFs (*n* = 4) at d0 (without treatment), d1 (with treatment), and d10 (post-treatment).

Supplementary Dataset S3B: Overlapping concordantly methylated genes in AA and EA TAS and CAFs. 168 overlapping concordantly methylated genes with the same direction of methylation in AA *vs.* EA in both TAS and CAFs identified. Methylation enrichment data provided for AA CAFs (*n* = 3) and EA CAFs (*n* = 4) at d0 (without treatment), d1 (with treatment), and d10 (post-treatment).

Supplementary Dataset S3C: Overlapping concordantly methylated genes with higher DNA

methylation levels in both AA TAS and AA CAFs. Of 168 genes, 109 show increased methylation levels in both AA TAS and AA CAFs. Methylation enrichment data provided for AA CAFs (n = 3) and EA CAFs (n = 4) at d0 (without treatment), d1 (with treatment), and d10 (post-treatment).

Supplementary Figure S1. A) Example of tumor and tumor adjacent stroma election. The red line indicates the approximate boundary between tumor and tumor adjacent stroma. **B)** Representative morphology of primary cultured CAF early in passage 6. **C)** Western blot analysis of α-smooth muscle actin (α-SMA) and vimentin (fibroblast markers), and tubulin (housekeeping gene) in African American (AA) (n = 4) and European American (EA) (n = 4) CAF primary cultures at passage 6. The image shows the entire uncropped blot with all visible bands and molecular weight markers. **D)** ImageJ software was used to calculate the ratio of α-SMA and vimentin to the levels of housekeeping gene (i.e., tubulin) , presented as bar graphs.

S1A

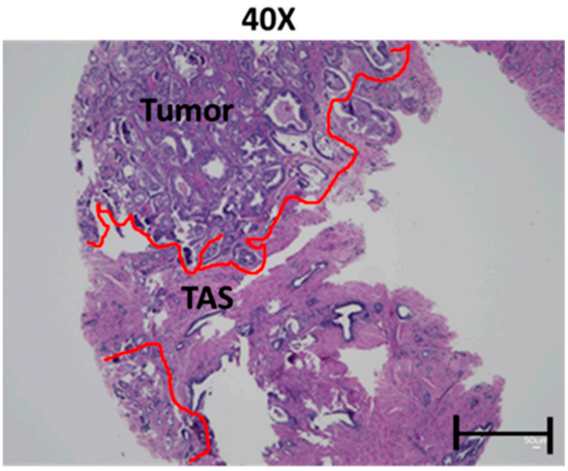

S1B

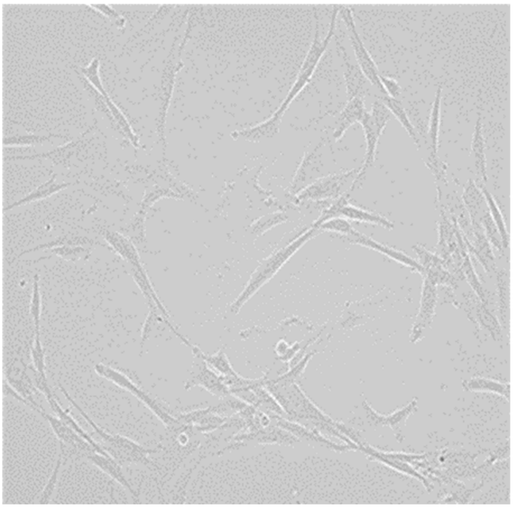

S1C

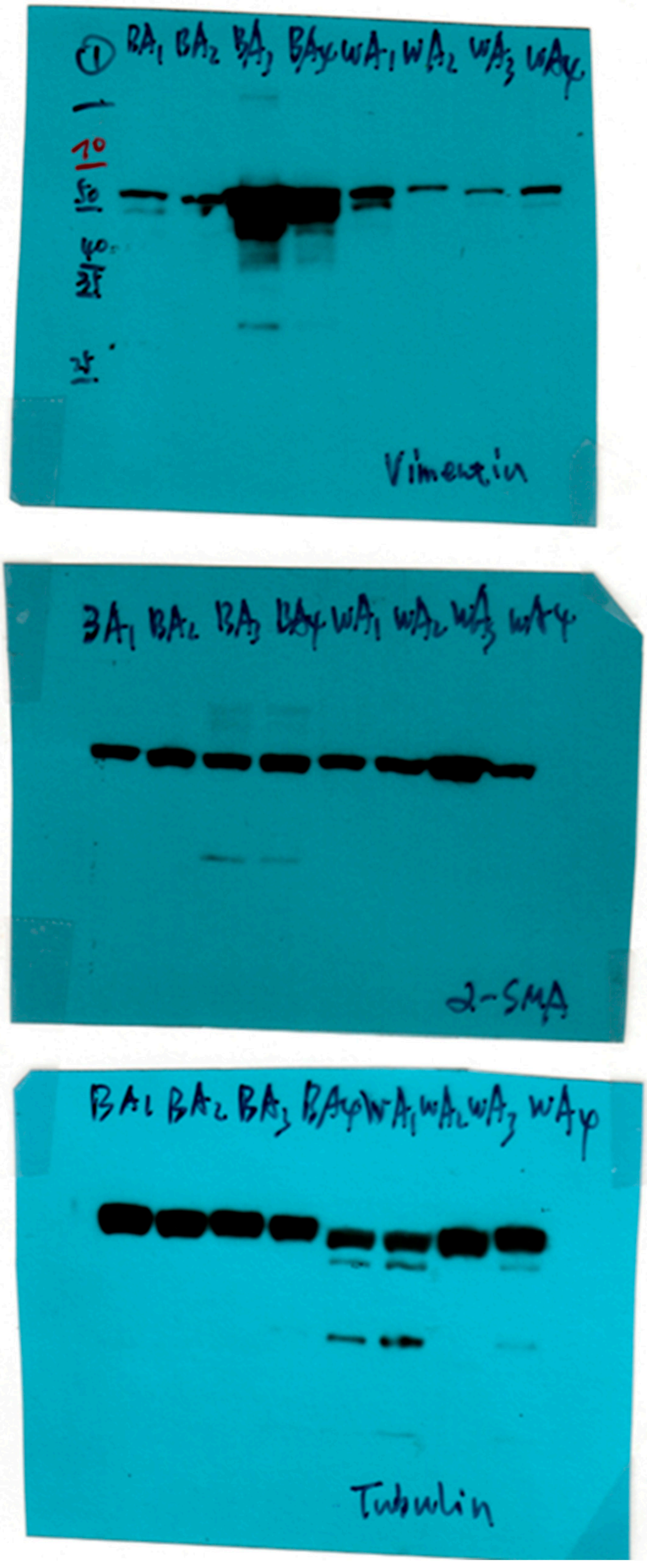
BA1 BA2 BA3 BA4 WA1 WA2 WA3 WA4
Vimentin
BA1 BA2 BA3 BA4 WA1 WA2 WA3 WA4
α-SMA
BA1 BA2 BA3 BA4 WA1 WA2 WA3 WA4
Tubulin

S1D

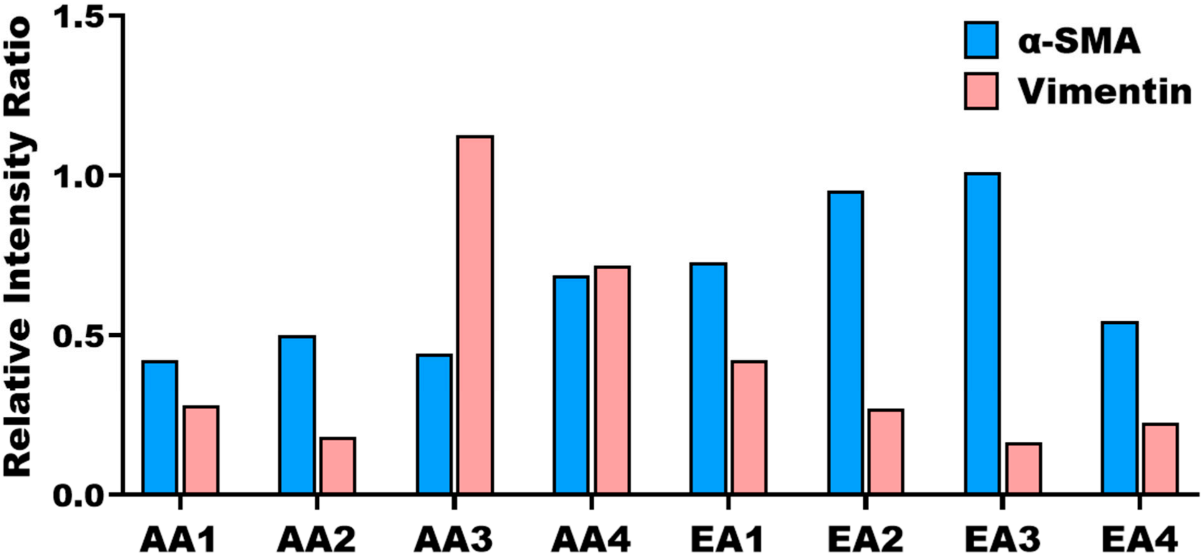

1.5
1.0
0.5
0.0
Relative Intensity Ratio
α-SMA
Vimentin
AA1
AA2
AA3
AA4
EA1
EA2
EA3
EA4
